# Mathematical Modeling of 6‑APA Production by Enzymatic Hydrolysis of Penicillin G in a Batch Reactor

**DOI:** 10.1021/acsomega.6c01501

**Published:** 2026-10-09

**Authors:** Letícia Eduarda Alves e Álvares, Lucas Figueiredo Formigosa, Marcos Vinícius da Silva Paula, Mailson Batista de Vilhena, Luciana Rocha Barros Gonçalves, Emanuel Negrão Macêdo, Bruno Marques Viegas

**Affiliations:** † Graduate Program in Biotechnology, 37871 Federal University of Pará, 66075-110 Belém, Pará, Brazil; ‡ Graduate Program in Chemical Engineering, 37871 Federal University of Pará, 66075-110 Belém, Pará, Brazil; § Graduate Program in Materials Science and Engineering, Federal University of Pará, 66075-110 Belém, Pará, Brazil; ∥ Department of Production Engineering, 681196 State University of Amapá, 68900-070 Macapá, Amapa, Brazil; ⊥ Department of Chemical Engineering, Federal University of Ceará, 60455-760 Fortaleza, Ceará, Brazil

## Abstract

6-Aminopenicillanic acid (6-APA) is a β-lactam intermediate of major industrial relevance and a key precursor for producing semisynthetic antibiotics. This study presents the development and analysis of a mathematical model describing 6-APA production via heterogeneous enzymatic hydrolysis of penicillin G in a batch reactor catalyzed by penicillin G acylase (PGA) immobilized on spherical supports. The model is formulated using coupled mass balances in the liquid and solid phases, explicitly considering diffusive and convective transport effects on the reaction kinetics. The resulting system of differential equations is solved using three independent approaches: the method of lines (MOL), adopted as the reference solution with explicit spatial dependence, the classical approximation, and the coupled integral equation approach with *H*_1,1_ approximation, the latter two being considered approximate solutions. A convergence analysis defines the spatial discretization required for the MOL solution. Comparison among the three methodologies shows excellent agreement, demonstrating the consistency of the solutions obtained. The results indicate that the mass Biot number (*Bi*_m_) plays a critical role in reactor performance, with complete conversion being achieved at reduced reaction times for *Bi*_m_ equal to 10. The results demonstrate the validity of the proposed model and the effectiveness of the solution methods used, contributing to the analysis and optimization of heterogeneous enzymatic processes at the industrial scale.

## Graphical abstract

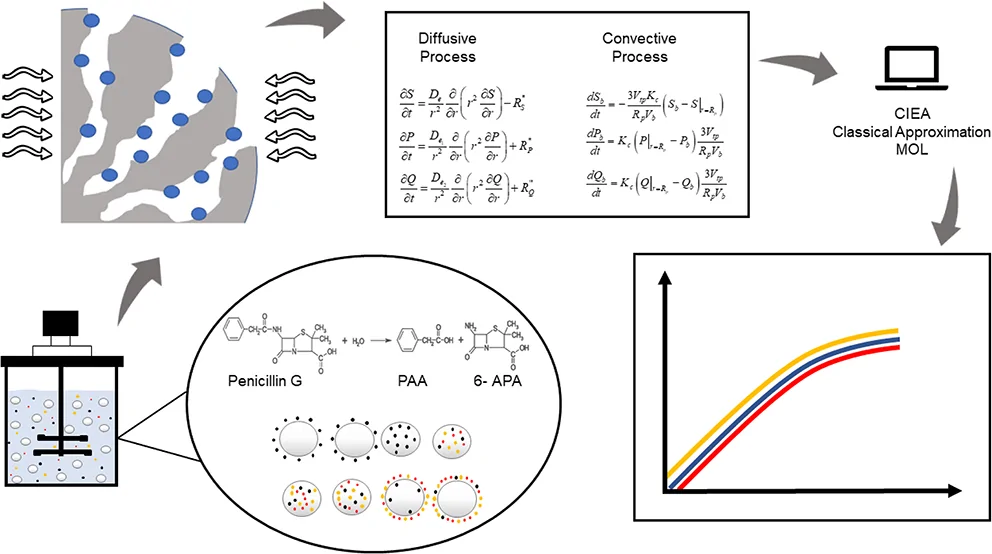

## 1 Introduction

6-Aminopenicillanic acid (6-APA) is a key β-lactam intermediate used in the synthesis of semisynthetic penicillins, and its discovery enabled the development of other β-lactam antibiotics of broad therapeutic relevance.^1,2^ β-Lactam antibiotics constitute a class of bactericidal drugs characterized by the presence of a β-lactam ring in their molecular structure, which is responsible for their antimicrobial activity.^3^ 6-APA is produced from penicillin G or V by enzymatic side-chain hydrolysis. This biocatalytic route has been increasingly investigated because of its economic and environmental advantages over the conventional chemical process, including fewer reaction steps and reduced waste generation.^4,5^ This process, an enzymatic hydrolysis catalyzed by penicillin G acylase (PGA), is governed by both kinetic and thermodynamic parameters that directly influence reactor performance.^6,7^

Penicillin G acylase (PGA) is an amidase belonging to the hydrolase class and exhibits reversible catalytic activity toward both acylation and deacylation reactions. The enzyme is widely used in the industrial production of β-lactam antibiotics, enabling the generation of key nuclei such as 6-APA and 7-aminodeacetoxycephalosporanic acid (7-ADCA) and the subsequent coupling with appropriate side chains.^5^ Under suitable conditions, PGA also catalyzes the reverse acylation reaction, in which activated acyl groups condense with 6-APA to form semisynthetic antibiotics, including amoxicillin and ampicillin.^8^

Enzyme immobilization on solid supports is a key requirement for the industrial implementation of enzymatic hydrolysis,^9,10^ as it provides several advantages over free enzymes. These include improved chemical and thermal stability, protection of the enzyme against denaturation, and biocatalyst reusability while maintaining catalytic activity over extended operation.^11^

Despite its industrial relevance, mathematical modeling of 6-APA production remains relatively underexplored in literature. Such models are important for kinetic parameter estimation, process monitoring, and analysis of key variables in penicillin G hydrolysis, including substrate concentration and product formation.^12^ Moreover, modeling supports the transition from laboratory to industrial operation by enabling process prediction and optimization under different operating conditions.^6,13^

However, previous studies have rarely proposed heterogeneous models for enzymatic systems that simultaneously describe transport in the liquid phase and within the immobilized particle, particularly those incorporating both external convection and intraparticle conversion of penicillin G. This limitation prevents an adequate description of the coupled diffusion–convection–reaction phenomena that govern immobilized biocatalyst performance and reactor behavior.

The heterogeneous enzymatic hydrolysis of penicillin G involves coupled diffusion, convection, and reaction phenomena, whose proper representation is essential for analyzing the performance of immobilized catalytic systems. In this work, a transient solid–liquid coupled model is developed that incorporates these transport mechanisms and enables evaluation of the influence of the mass Biot number (*Bi*_m_) on the overall reaction kinetics. The formulation predicts the temporal evolution of concentrations in the bulk and within the catalytic particle, providing a physically consistent description of the system behavior.

The proposed mathematical model is based on a common set of mass balances, kinetic mechanism, physical assumptions, and boundary conditions. Three approaches are considered to obtain its solution: the method of lines (MOL), the classical approximation, and the coupled integral equation approach (CIEA). MOL explicitly retains the spatial dependence of the partial differential equations through domain discretization and is adopted as the reference solution.^14,15^ The classical approximation provides an approximate solution by representing the concentrations within the immobilized particle by their volume-averaged values.^16^ The CIEA also provides an approximate solution by combining integral equations with Hermite approximations to retain boundary information and approximately represent concentration variations within the particles.^17,18^ Comparison of the results obtained using these three approaches allows the approximate solutions to be evaluated against the MOL reference solution.

The main contribution of this work is the development of a transient coupled solid–liquid mathematical model for heterogeneous enzymatic systems that integrates convection, diffusion, and reaction phenomena within a unified framework. Unlike simplified formulations that neglect spatial concentration variations, the proposed model explicitly describes penicillin G conversion in both the bulk liquid phase and the catalytic particle, providing a detailed representation of the transport–reaction interactions governing the system. This formulation enables systematic evaluation of mass-transfer effects on the overall reaction kinetics and provides a physically consistent framework for the analysis of heterogeneous enzymatic reactors. The proposed mathematical model is applied to the enzymatic hydrolysis of penicillin G catalyzed by immobilized PGA in a batch reactor, with the three solution approaches compared in terms of accuracy and computational efficiency. MOL has been widely applied to heat transfer,^19^ diffusion,^20^ and convection problems, while the classical approximation and the CIEA with *H*_1,1_ approximation have also been applied to the solution of diffusion-reaction problems.^21–23^

## 2 Materials and Methods

### 2.1 Mathematical Modeling

A mathematical model was developed for the enzymatic hydrolysis of penicillin G catalyzed by PGA to describe the formation of the two main products, 6-aminopenicillanic acid (6-APA) and phenylacetic acid (PAA). The formulation was based on mass balances for a heterogeneous batch reactor comprising two phases: a liquid phase, where the substrate and products were dissolved, and a solid phase consisting of particles containing the immobilized enzyme. A schematic representation of the reaction system is shown in Figure 1.

**Figure 1:**
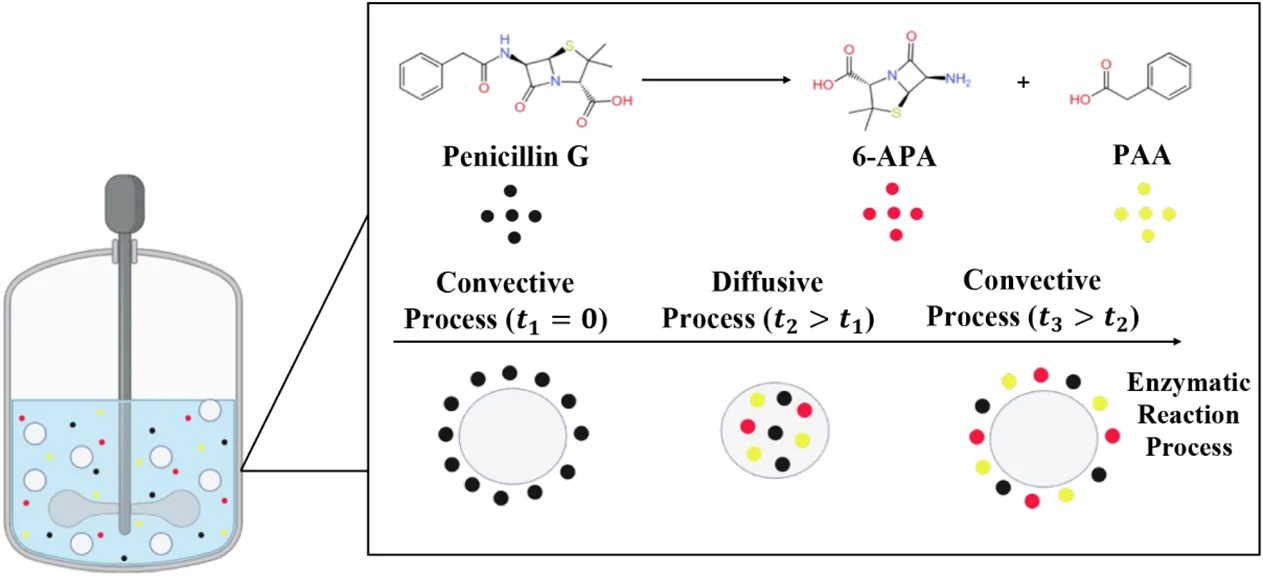
Schematic representation of a heterogeneous batch reactor with an immobilized enzyme to produce 6-APA and PAA.

The mathematical model was formulated under the following assumptions: (1) substrate and product concentrations vary with time and along the particle radial coordinate, and the system is described by a one-dimensional transient model; (2) the effective diffusion coefficients of all species are constant; (3) the reactor operates in batch mode; (4) temperature and pH remain constant throughout the reaction; (5) biocatalyst particles are uniformly dispersed in the reactor bulk; (6) enzyme thermal deactivation is neglected over the reaction time; (7) the liquid phase contains dissolved substrate and products only; (8) the enzymatic reaction occurs exclusively within the solid phase of the biocatalyst; (9) substrate is transferred from the liquid phase into the particles, while products diffuse from the particle interior to the bulk liquid; and (10) the kinetic mechanism includes competitive inhibition by PAA and noncompetitive inhibition by 6-APA and penicillin G.

#### 2.1.1 Mass Balance in the Bulk Phase

The formulation of the mathematical model was based on the application of mass balances to the chemical species present in the bulk phase of the reactor. Figure 2 illustrates the reaction system operating in batch mode, in which the substrate *C*_S_ encountered the enzyme (*C*_E_) immobilized in the solid phase. This enzyme catalyzed the conversion of the substrate, resulting in the formation of 6-APA, represented in the model by *C*_P_, and PAA, represented by *C*_Q_.

**Figure 2:**
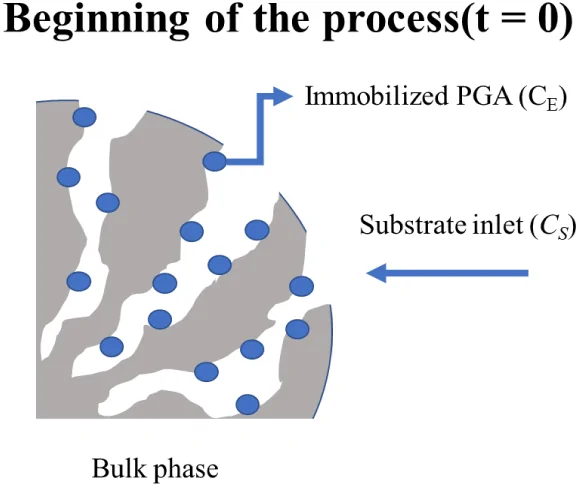
Schematic representation of the batch reactor system at the initial time (*t* = 0).

The mass balance illustrated in Figure 2 was described by a set of differential equations accounting for the rates of consumption and formation of the chemical species in the system, representing the behavior of the species in the bulk phase. In a reactor operating in batch mode, the substrate concentration varied with time. In the bulk phase, this concentration decreased progressively due to the diffusion of the substrate into the catalytic particles, where the enzymatic hydrolysis responsible for product formation occurred.

The consumption of substrate *C*_S_ was governed by the reaction kinetics of the enzymatic system and was described by eq 1. In this formulation, the bulk-phase volume (*V*_b_) was assumed to remain constant throughout the process. The total surface area of the particles (*A*_p_) was defined as a function of the total particle volume (*V*_tp_) and the corresponding particle radius (*R*_p_), while *K*_c_ represents the external convective mass-transfer coefficient between the phases. The complete derivation of this equation from the mass balance is provided in the eqs S1–S6 in the Supporting Information.

1 $$\frac{dC_{\mathrm{S,b}}}{dt}=-\frac{3V_{\mathrm{tp}}K_{c}}{R_{p}V_{b}}\left(C_{\mathrm{S,b}}-{C_{S}|}_{r=R_{p}}\right)$$

Similarly, the mass balances and their respective initial conditions for products *C*_P_ and *C*_Q_ were described by eqs 2 and 5 for *C*_P_ and by eqs 3 and 6 for *C*_Q_, since product formation occurred inside the catalytic particles. Equation 4 represents the initial condition for the substrate in the bulk phase, where *C*_S,b_ denotes the concentration of *C*_S_ in the bulk phase and $C_{\mathrm{S,b}}^{0}$ denotes the initial concentration of *C*_S_ in the bulk phase. Similarly, *C*_P,b_ and *C*_Q,b_ denotes the concentrations of the products *C*_P_ and *C*_Q_ in the bulk phase, respectively.

2 $$\frac{dC_{\mathrm{P,b}}}{dt}=K_{c}\left({C_{P}|}_{r=R_{p}}-C_{\mathrm{P,b}}\right)\frac{3V_{\mathrm{tp}}}{R_{p}V_{b}}$$

3 $$\frac{dC_{\mathrm{Q,b}}}{dt}=K_{c}\left({C_{Q}|}_{r=R_{p}}-C_{\mathrm{Q,b}}\right)\frac{3V_{\mathrm{tp}}}{R_{p}V_{b}}$$

At *t* = 0, the values of *C*_S_, *C*_P_, and *C*_Q_ are the following:

4 $$C_{\mathrm{S,b}}=C_{\mathrm{S,b}}^{0}$$

5 $$C_{\mathrm{P,b}}=0$$

6 $$C_{\mathrm{Q,b}}=0$$

#### 2.1.2 Mass Balance in the Solid Phase

The mass balance formulated for the catalytic particle considered the simultaneous occurrence of diffusion and chemical reaction, describing the temporal and spatial variation of the concentrations of the reacting species, namely the substrate and the products. Assuming the absence of convection within the solid phase and that mass transport occurred predominantly along the radial coordinate, eq 7 was obtained. The complete derivation of this equation is presented in the Supporting Information, eqs S7–S10. The resulting expression relates the concentration profile inside the particle to the combined effects of intraparticle diffusion and reaction.

7 $$\frac{\partial C_{i}}{\partial t}=\frac{1}{r^{2}}\frac{\partial }{\partial r}\left(r^{2}D_{i}\frac{\partial C_{i}}{\partial r}\right)\pm R_{i}$$

in which *C*_i_ represents the concentration of species i (S, P, or Q) in the solid phase, *r* is the radial coordinate, *D*_i_ is the effective diffusivity of species i, and *R*_i_ is the rate of substrate consumption or product formation.

The application of eq 7 to the substrate *C*_S_ and the products *C*_P_ and *C*_Q_, together with the corresponding kinetic rate expressions, yields the system of differential equations given by eqs 8–16. The formulation assumes constant effective diffusivities, namely, *D*_e_ for the substrate, *D*_e1_ for product *C*_P_, and *D*_e2_ for product *C*_Q_. Initially (*t* = 0), the concentrations of substrate and products within the catalytic particle are assumed to be zero, as represented by eqs 11–13. At the center of the spherical particle (*r* = 0), symmetry boundary conditions are imposed, resulting in zero concentration gradients for all species, as given by eqs 14–16.

8 $$\frac{\partial C_{S}}{\partial t}=\frac{D_{e}}{r^{2}}\frac{\partial }{\partial r}\left(r^{2}\frac{\partial C_{S}}{\partial r}\right)-R_{C_{S}}$$

9 $$\frac{\partial C_{P}}{\partial t}=\frac{D_{\mathrm{e1}}}{r^{2}}\frac{\partial }{\partial r}\left(r^{2}\frac{\partial C_{P}}{\partial r}\right)+R_{C_{P}}$$

10 $$\frac{\partial C_{Q}}{\partial t}=\frac{D_{\mathrm{e2}}}{r^{2}}\frac{\partial }{\partial r}\left(r^{2}\frac{\partial C_{Q}}{\partial r}\right)+R_{C_{Q}}$$

11 $$C_{P}=0$$

12 $$C_{S}=0$$

13 $$C_{Q}=0$$

14 $$\frac{\partial C_{P}}{\partial t}=0$$

15 $$\frac{\partial C_{S}}{\partial t}=0$$

16 $$\frac{\partial C_{Q}}{\partial t}=0$$

in which $R=R_{C_{S}}=R_{C_{P}}=R_{C_{Q}}$ denotes the rate of enzymatic hydrolysis of penicillin G, as proposed by Kheirolomoom et al.^24^ and given by eq 17. This kinetic expression is based on the Michaelis–Menten formalism and incorporates substrate inhibition, competitive inhibition by 6-APA, and noncompetitive inhibition by phenylacetic acid. The parameters *K*, *K*_S_, *K*_1_, and *K*_2_ represent the Michaelis–Menten, substrate inhibition, competitive inhibition, and noncompetitive inhibition constants, respectively, while *K*_r_ denotes the intrinsic catalytic rate constant of the enzymatic reaction.

17 $$R=\frac{K_{r}C_{E}C_{S}}{K\left[1+\frac{C_{S}}{K}+\frac{{C_{S}}^{2}}{KK_{S}}+\frac{C_{P}}{K_{1}}+\frac{C_{Q}}{K_{2}}+\frac{C_{S}C_{Q}}{KK_{2}}+\frac{C_{P}C_{Q}}{K_{1}K_{2}}\right]}$$

The reaction mechanism for the hydrolysis of penicillin G catalyzed by PGA is presented in Figure 3. Part (a) illustrates the classical Michaelis–Menten mechanism, in which the enzyme (E) reversibly binds the substrate (S) to form the enzyme–substrate complex (ES), according to E + S ⇄ ES. The formation and dissociation of this complex are governed by the rate constants *k*_1_ and *k*_–1_, respectively. The ES complex is subsequently converted into products through the rate constant *k*_2_. Based on the Briggs–Haldane steady-state assumption, the rate of ES formation is equal to its rate of consumption, leading to the definition of the Michaelis–Menten constant *K*.^25–27^ Part (b) represents competitive inhibition by 6-APA (P), where the enzyme interacts with the inhibitor to form the inactive complex EP, characterized by the inhibition constant *K*_1_. Part (c) describes excess substrate inhibition through the formation of the inactive complex ESS, governed by the constant *K*_S_. Part (d) illustrates noncompetitive inhibition by phenylacetic acid (Q), where the enzyme forms the inactive complex EQ, represented by the inhibition constant *K*_2_.

**Figure 3:**
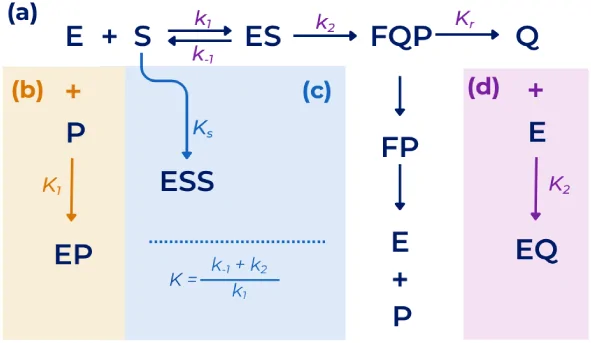
Schematic representation of the reaction mechanism for the enzymatic hydrolysis of penicillin G.

#### 2.1.3 Boundary Condition at the Particle Surface (*R* = *R*_p_)

At the particle surface, continuity of mass flux between the phases was assumed; therefore, the diffusive flux inside the particle equals the convective mass transfer flux in the liquid phase. No chemical reaction was considered at the particle surface, as illustrated in Figure 4. This boundary condition represents conservation of mass at the solid–liquid interface.

**Figure 4:**
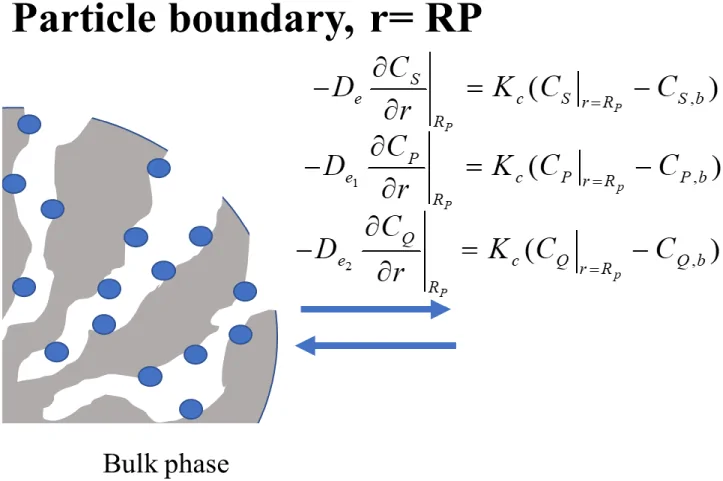
Mass transfer at the particle surface.

The application of this boundary condition to the substrate leads to eq 18. Similarly, its application to the products yields eq 19 for *C*_P_ and eq 20 for *C*_Q_.

18 $$-D_{e}{\frac{\partial C_{S}}{\partial r}|}_{R_{p}}=K_{c}\left({C_{S}|}_{r=R_{p}}-C_{\mathrm{S,b}}\right)$$

19 $$-D_{\mathrm{e1}}{\frac{\partial C_{P}}{\partial r}|}_{R_{p}}=K_{c}\left({C_{P}|}_{r=R_{p}}-C_{\mathrm{P,b}}\right)$$

20 $$-D_{\mathrm{e2}}{\frac{\partial C_{Q}}{\partial r}|}_{R_{p}}=K_{c}\left({C_{Q}|}_{r=R_{p}}-C_{\mathrm{Q,b}}\right)$$

### 2.2 Dimensionless Formulation

Equations 21–40 define the dimensionless groups employed in this study, which allow the analysis of the relative importance of diffusion, convection, and reaction in the system.

The parameters α, α_S_, α_P_ and α_Q_ denote the dimensionless kinetic constants associated with the Michaelis–Menten constant and the substrate and product inhibition constants, respectively. The parameters γ_S_, γ_P_ and γ_Q_ correspond to the effective diffusivities of the substrate and products normalized by the reference diffusivity *D*_*r*_. The variable η denotes the dimensionless radial coordinate, whereas $C_{S}^{*}$ , $C_{P}^{*}$ , and $C_{Q}^{*}$ are the dimensionless concentrations of the substrate and products within the solid phase. Similarly, $C_{\mathrm{S,b}}^{*}$ , $C_{\mathrm{P,b}}^{*}$ , and $C_{\mathrm{Q,b}}^{*}$ denote the corresponding dimensionless concentrations in the liquid phase. The parameter τ represents the dimensionless time, i.e., *t* normalized by the characteristic time *T*_r_, while φ_part_ corresponds to the particle volume fraction in the reactor.

21 $$\alpha =\frac{C_{\mathrm{S,b}}^{0}}{K}$$

22 $$\alpha_{S}=\frac{C_{\mathrm{S,b}}^{0}}{K_{S}}$$

23 $$\alpha_{P}=\frac{C_{\mathrm{S,b}}^{0}}{K_{1}}$$

24 $$\alpha_{Q}=\frac{C_{\mathrm{S,b}}^{0}}{K_{2}}$$

25 $${Bi}_{m}=\frac{K_{c}R_{p}}{D_{r}}$$

26 $$\gamma_{S}=\frac{D_{e}}{D_{r}}$$

27 $$\gamma_{P}=\frac{D_{\mathrm{e1}}}{D_{r}}$$

28 $$\gamma_{Q}=\frac{D_{\mathrm{e2}}}{D_{r}}$$

29 $$\eta =\frac{r}{R_{p}}$$

30 $$C_{S}^{*}=\frac{C_{S}}{C_{\mathrm{S,b}}^{0}}$$

31 $$C_{P}^{*}=\frac{C_{P}}{C_{\mathrm{S,b}}^{0}}$$

32 $$C_{Q}^{*}=\frac{C_{Q}}{C_{\mathrm{S,b}}^{0}}$$

33 $$\tau =\frac{t}{T_{r}}$$

34 $$T_{r}=\frac{{R_{p}}^{2}}{D_{r}}$$

35 $$C_{\mathrm{S,b}}^{*}=\frac{C_{\mathrm{S,b}}}{C_{\mathrm{S,b}}^{0}}$$

36 $$C_{\mathrm{P,b}}^{*}=\frac{C_{\mathrm{P,b}}}{C_{\mathrm{S,b}}^{0}}$$

37 $$C_{\mathrm{Q,b}}^{*}=\frac{C_{\mathrm{Q,b}}}{C_{\mathrm{S,b}}^{0}}$$

38 $$\varphi_{\mathrm{part}}=\frac{V_{\mathrm{tp}}}{V_{t}}$$

39 $$\frac{V_{b}}{V_{t}}=\frac{\varphi_{\mathrm{part}}}{\left(1-\varphi_{\mathrm{part}}\right)}$$

40 $$\psi =\frac{R_{p}}{3}\sqrt{\frac{K_{r}C_{E}}{KD_{r}}}$$

For the nondimensionalization of the reaction rate, the terms in eq 17 were replaced with their corresponding dimensionless forms. Following this substitution, the resulting expression was multiplied by *T*_r_/C_S,b_^0^, which, after algebraic manipulation, yielded eqs 41–43. The dimensionless reaction rate is given by eq 41:

41 $$R^{*}=9\Psi 2R^{\prime \prime \prime }$$

in which *R*‴ is the reaction term given by eq 42 and Ψ is the Thiele modulus, given by eq 43:

42 $$R^{\prime \prime \prime }=C_{S}^{*}/\left(1+\alpha_{S}C_{S}^{*}+\alpha \alpha_{S}{\left(C_{S}^{*}\right)}^{2}+\alpha_{P}C_{P}^{*}+\alpha_{Q}C_{Q}^{*}+\alpha \alpha_{Q}C_{S}^{*}C_{Q}^{*}+\alpha_{P}\alpha_{Q}C_{P}^{*}C_{Q}^{*}\right)$$

43 $$\Psi =\frac{R_{p}}{3}\sqrt{\frac{k_{r}C_{E}}{KD_{e}}}$$

### 2.3 Model Solution Methodology

The dimensionless system of differential equations, derived from a single physical model, was solved using three different approaches. MOL was adopted as the reference solution, retaining the spatial description through domain discretization, whereas the classical approximation and CIEA-*H*_1,1_ provide approximate solutions based on simplified representations of the concentration profiles within the immobilized particles.

Finite difference approximations are commonly derived from the Taylor series expansion, allowing derivatives to be estimated from surrounding nodal values while providing a direct assessment of the associated truncation error.^28^ Different approximation schemes can be obtained according to the number and spatial distribution of the selected nodes. The three-point forward and backward approximations for the first derivative are given by eqs 44 and 45, respectively:

44 $$f^{\prime }\left(C_{i,j}^{*}\right)=\frac{-3f\left(C_{i,j}^{*}\right)+4f\left(C_{i,j+1}^{*}\right)-f\left(C_{i,j+2}^{*}\right)}{2\Delta \eta }$$

45 $$f^{\prime }\left(C_{i,j}^{*}\right)=\frac{f\left(C_{i,j-2}^{*}\right)-4f\left(C_{i,j-1}^{*}\right)-3f\left(C_{i,j}^{*}\right)}{2\Delta \eta }$$

The central finite difference approximations for the first and second derivatives are given by eqs 46 and 47, respectively:

46 $$f^{\prime }\left(C_{i,j}^{*}\right)=\frac{f\left(C_{i,j+1}^{*}\right)-f\left(C_{i,j-1}^{*}\right)}{2\Delta \eta }$$

47 $$f^{\prime \prime }\left(C_{i,j}^{*}\right)=\frac{f\left(C_{i,j-2}^{*}\right)-2f\left(C_{i,j-1}^{*}\right)+f\left(C_{i,j}^{*}\right)}{\Delta \eta^{2}}$$

in which i represents the components (S, P, and Q) and *j* the mesh points.

The classical approximation corresponds to a lumped formulation of the governing equations, in which the spatial distribution of concentration within the particle is replaced by its volume-averaged value. This simplification is appropriate when the particle diameter is sufficiently small and internal concentration gradients can be neglected. Under these conditions, the original partial differential equations are reduced to a system of ordinary differential equations describing the temporal evolution of the average concentrations. Because of its simplified structure, this formulation provides a useful reference for comparison with the more detailed distributed parameter models and for assessing the consistency of the predicted results.^29–31^ The resulting equations are given by eqs 48–50:

48 $$C_{S}\left(\eta ,\tau \right)\approx {\mathrm{C̃}}_{S}\left(\tau \right)$$

49 $$C_{P}\left(\eta ,\tau \right)\approx {\mathrm{C̃}}_{P}\left(\tau \right)$$

50 $$C_{Q}\left(\eta ,\tau \right)\approx {\mathrm{C̃}}_{Q}\left(\tau \right)$$

The CIEA is a reformulation framework in which the governing partial differential equations are transformed into a coupled system containing differential and integral representations of the original problem. This formulation combines local and integral information, preserving the main features of the spatial distribution while reducing the complexity associated with the direct solution of the original equations. The resulting system is obtained through integral balances and the evaluation of derivatives at the integration boundaries.^21,22,31^ In the present work, Hermite approximations were adopted to evaluate the integral terms appearing in the CIEA formulation. The general Hermite approximation is given by eq 51:^18^

51 $$\int_{x_{i-1}}^{x_{i}}y\left(x\right)\;dx\approx \overset{\alpha_{k}}{\underset{v=0}{\sum }}C_{v}y_{i-1}^{\left(v\right)}+\overset{\beta }{\underset{v=0}{\sum }}D_{v}y_{i}^{\left(v\right)}$$

in which *y*(*x*) and its derivatives *y* ^(*v*)^(*x*) are defined for all *x* ∈ (*x_i_*_–1_, *x_i_*). The formulation assumes that the values of the function and its derivatives are available at the interval end points, namely, *y* ^(*v*)^(*x*_*i*–1_) = *y*_*i*–1_^(*v*)^ for *v* = 0, 1, 2, ..., α*_k_* and *y* ^(*v*)^(*x*) = *y*_*i*_^(*v*)^ for *v* = 0, 1, 2, ..., β. Under these conditions, the integral of *y*(*x*) over the interval can be approximated as a linear combination of the function values and derivative values at the integration boundaries. This family of Hermite approximations is denoted by $H_{\alpha_{k},\beta }$ , where α*_k_* and β indicate the highest derivative orders retained at the lower and upper integration limits, respectively.^17,32^ The general expression for the $H_{\alpha_{k},\beta }$ approximation is given by eq 52:

$$\int_{x_{i-1}}^{x_{i}}y\left(x\right)\;dx=\overset{\alpha_{k}}{\underset{v=0}{\sum }}C_{v}(\alpha_{k},\beta )\times (-1v)\times h_{i}^{v+1}y_{i-1}^{\left(v\right)}+\overset{\beta }{\underset{v=0}{\sum }}C_{v}(\beta ,\alpha_{k})\times (-1v)\times h_{i}^{v+1}y_{i}^{\left(v\right)}$$ 52

in which *h*_*i*_ denotes the interval length, defined by eq 53, and *C*_*v*_(α_*k*_,β) is the coefficient associated with the $H_{\alpha_{k},\beta }$ approximation, given by eq 54:

53 $$h_{i}=x_{i}-x_{i-1}$$

$$C_{v}\left(\alpha_{k},\beta \right)=\frac{\left(\alpha_{k}+1\right)!\left(\alpha_{k}+\beta +1-v\right)!}{\left(v+1\right)!\left(\alpha_{k}-v\right)!\left(\alpha_{k}+\beta +2\right)!}$$ 54

The most used Hermite approximations are *H*_0,0_ (trapezoidal rule) and *H*_1,1_ (corrected trapezoidal rule), given by eqs 55 and 56, respectively:

55 $$H_{0,0}=\int_{0}^{h}y\left(x\right)\;dx\approx \frac{h}{2}\left[y\left(0\right)+y\left(h\right)\right]$$

56 $$H_{1,1}=\int_{0}^{h}y\left(x\right)\;dx\approx \frac{h}{2}\left[y\left(0\right)+y\left(h\right)\right]+\frac{h^{2}}{12}[y^{\prime }\left(0\right)-y^{\prime }\left(h\right)]$$

While the *H*_0,0_ approximation is based only on function values at the integration boundaries, the *H*_1,1_ approximation also incorporates first-order derivative information at both limits of the interval. Consequently, *H*_1,1_ provides a more accurate representation of the integral terms and the associated concentration gradients, leading to improved approximations of concentration profiles and fluxes.^31,32^ For this reason, the *H*_1,1_ approximation was adopted in the present work.

The use of the three formulations, namely MOL, the classical approximation, and CIEA-*H*_1,1_, enables the comparison of distributed and lumped formulations of the diffusion-reaction problem and provides an additional verification of the consistency of the predicted results. The mathematical development leading to the final equations for these formulations, as well as the corresponding equations for the reaction products, is presented in detail in eqs S11–S90.

#### 2.3.1 Method of Lines

In this method, the spatial coordinates are discretized, transforming the partial differential equations (PDEs) into a system of ordinary differential equations (ODEs) while the time variable remains continuous.^33–35^ This characteristic acknowledges it as particularly suitable for problems involving mass transfer and chemical reactions with complex boundary conditions.^36,37^

The spatial domain along the dimensionless radial coordinate (η) was discretized using a uniform mesh, in which the number of grid points (*J*) determines the spacing between nodes. Higher values of *J* lead to increased spatial resolution and, consequently, greater numerical accuracy. The discretized mass balance equations for the substrate are given in eqs 57–62, including the formulations for the solid and bulk phases, together with the corresponding boundary conditions at η = 0 and η = 1 and the initial conditions at τ = 0.

57 $$\frac{dC_{S}^{*}}{d\tau }=\gamma_{S}\left(\frac{2}{\left(j-1\right)\Delta \eta }\frac{C_{S,J+1}^{*}-C_{S,J-1}^{*}}{2\Delta \eta }+\frac{1}{\Delta \eta^{2}}C_{S,J+1}^{*}-2C_{S,J}^{*}+C_{S,J-1}^{*}\right)-R_{m}^{*}$$

58 $$\frac{dC_{\mathrm{S,b}}^{*}}{d\tau }=3\frac{{Bi}_{m}\varphi_{\mathrm{part}}\left(-C_{\mathrm{S,b}}^{*}+C_{S}^{*}\right)}{1-\varphi_{\mathrm{part}}}$$

59 $$C_{S,0}^{*}=\frac{-3C_{S,0}^{*}+4C_{S,1}^{*}-C_{S,2}^{*}}{2\Delta \eta }=0$$

60 $$C_{S,Ni}^{*}=-\gamma_{S}\frac{C_{S,N_{i}-2}^{*}-4C_{S,N_{i}-1}^{*}+C_{S,N_{i}}^{*}}{2\Delta \eta }$$

61 $$C_{S}^{*}=0$$

62 $$C_{\mathrm{S,b}}=1$$

#### 2.3.2 Classical Approach

The classical approximation, formulated in terms of lumped parameters, constitutes the simplest approach for solving the model, as widely reported in the literature.^19,29,30,38^ This methodology assumes that radial concentration gradients within the particle are negligible, allowing the surface concentrations to be approximated by their volume-averaged values. Under these assumptions, the governing equations for the substrate in both the solid and bulk phases are given in eqs 63–67, together with the corresponding initial conditions. In this formulation, *I* represents the dimensionless average reaction term associated with the classical approximation.

63 $$\frac{d{\mathrm{C̃}}_{S}^{*}}{d\tau }=-3{Bi}_{m}\left(C_{S}^{*}-C_{\mathrm{S,b}}^{*}\right)-3I_{S}^{*}$$

64 $$\frac{dC_{\mathrm{S,b}}^{*}}{d\tau }=3\frac{\varphi_{\mathrm{part}}}{1-\varphi_{\mathrm{part}}}{Bi}_{m}{\left(C_{\mathrm{S,b}}^{*}-C_{S}^{*}\right)|}_{\eta =1}$$

65 $$C_{\mathrm{S,b}}^{*}=C_{\mathrm{S,b}}^{*,0}$$

66 $$C_{S}^{*}=0$$

67 $$I^{*}=\frac{R_{\mathrm{av}}^{*}}{3}$$

In this formulation, the term $R_{\mathrm{av}}^{*}$ represents the average reaction rate associated with the classical approximation and, similarly, is defined with respect to the dimensionless reaction term presented in eqs 41 and 42.

#### 2.3.3 Coupled Integral Equation Approach

The lumped-parameter approximation can be improved using the CIEA, which enhances the representation of the concentration at the domain boundary by expressing it as a function of the average concentration within the medium, as proposed by Mikhailov and Cotta^31^ and Picanço and Macêdo.^39^ In this approach, both the flux integral and the domain integral are evaluated analytically and approximated using the Hermite formula of type *H*_1,1_.^18^ The main objective of the CIEA is to preserve the essential physical information on the phenomenon along the eliminated spatial direction, resulting in a more accurate description of concentration profiles and interfacial fluxes when compared with classical approximation.

After application of the CIEA, the governing equations for the substrate are obtained for both the solid and bulk phases, together with the corresponding initial and boundary conditions at η = 0 and η = 1.

68 $$\frac{d{\mathrm{C̃}}_{S}^{*}}{d\tau }=-3{Bi}_{m}\left(C_{S}^{*}-C_{\mathrm{S,b}}^{*}\right)-3I_{S}^{*}$$

69 $$\frac{dC_{\mathrm{S,b}}^{*}}{d\tau }=3\frac{\varphi_{\mathrm{part}}}{1-\varphi_{\mathrm{part}}}{Bi}_{m}{\left(C_{\mathrm{S,b}}^{*}-C_{S}^{*}\right)|}_{n=1}$$

70 $$C_{\mathrm{S,b}}^{*}=C_{\mathrm{S,b}}^{*,0}$$

71 $$C_{S}^{*}=0$$

72 $$\frac{d{\mathrm{C̃}}_{S,0}^{*}}{d\tau }={3\lambda_{S}\frac{\partial^{2}C_{S}^{*}}{\partial \eta^{2}}|}_{\eta =0}-{R_{i}^{*}|}_{\eta =0}$$

73 $$\frac{\partial C_{S,1}^{*}}{\partial \tau }=\left\{{2\frac{\partial C_{S}^{*}}{\partial \eta }|}_{\eta =1}+{\frac{\partial^{2}C_{S}^{*}}{\partial \eta^{2}}|}_{\eta =1}\right\}-{R_{i}^{*}|}_{\eta =1}$$

74 $$C_{\mathrm{S,b}}^{*}=C_{\mathrm{S,b}}^{*,0}$$

75 $$C_{S}^{*}=0$$

76 $$C_{S,0}^{*}=0$$

77 $$C_{S,1}^{*}=0$$

In this formulation, the integral term $I_{S}^{*}=I_{i}^{*}$ is defined as given in eq 78.

78 $$I_{i}^{*}=\frac{1}{3}{\mathrm{R̃}}_{\mathrm{av}}^{*}$$

### 2.4 Model Parameters and Initial Conditions

The parameters used in solving the mathematical model were obtained from Valencia et al.^40^ and are presented in Table 1. The initial substrate concentration in the bulk phase was set to 150 mM.

**Table 1:** Parameters for the Enzymatic Hydrolysis of Penicillin G

parameter	symbol	value
substrate effective diffusion coefficient (m^2^/s)	*D*_e_	5.3 × 10^–10^
effective diffusion coefficient of *C*_P_ (m^2^/s)	*D*_e1_	7.33 × 10^–10^
effective diffusion coefficient of *C*_Q_ (m^2^/s)	*D*_e2_	5.89 × 10^–10^
total particle volume (mL)	*V*_tp_	8.88 × 10^–8^
bulk phase volume (mL)	*V*_b_	40 × 10^–6^
intrinsic reaction rate constant (1/s)	*K*_r_	41
enzyme concentration (mM)	*C*_E_	0.2143
Michaelis–Menten constant for penicillin G (mM)	*K*	0.13
substrate inhibition constant (mM)	*K*_S_	821
inhibition constant for *C*_P_ (mM)	*K*_1_	1.82
inhibition constant for *C*_Q_ (mM)	*K*_2_	48

All numerical simulations were performed using the NDSolve function available in Wolfram Mathematica. For the classical approximation, temporal integration was carried out using the ImplicitRungeKutta method with DifferenceOrder set to 10, AccuracyGoal equal to 8, and PrecisionGoal equal to 8. For the CIEA-*H*_1,1_ formulation, the StiffnessSwitching method was adopted due to the stiff nature of the resulting system, with AccuracyGoal and PrecisionGoal both set to 8. For the MOL formulation, temporal integration was also performed using the StiffnessSwitching method, with AccuracyGoal and PrecisionGoal set to 7. These settings were selected to ensure that the temporal integration error remained compatible with the five-digit accuracy criterion adopted in the spatial discretization. Additional numerical tests demonstrated that further increases in the temporal integration accuracy did not produce changes in the reported results within five significant digits.

## 3 Results and Discussion

### 3.1 Convergence Analysis

The convergence analysis was performed to ensure that the numerical solutions obtained with the proposed method are independent of the spatial discretization, converging to stable values as the mesh is refined. Table 2 presents the results of the convergence analysis for the substrate concentration in the bulk phase.

**Table 2:** Convergence Analysis for the Substrate Concentration in the Bulk Phase (*C*_S,b_^*^)

*C*_S,b_^*^	τ	*N* = 10	*N* = 20	*N* = 30	*N* = 40	*N* = 50
*Bi*_m_ = 0.01	10	**0.9993**	**0.9993**	**0.9993**	**0.9993**	**0.9993**
	100	**0.9934**	**0.9934**	**0.9934**	**0.9934**	**0.9934**
	1000	**0.9362**	**0.9362**	**0.9362**	**0.9362**	**0.9362**
*Bi*_m_ = 0.1	10	0.9941	**0.9940**	**0.9940**	**0.9940**	**0.9940**
	100	**0.9475**	**0.9475**	**0.9475**	**0.9475**	**0.9475**
	1000	0.5755	**0.5754**	**0.5754**	**0.5754**	**0.5754**
*Bi*_m_ = 1	10	**0.9852**	**0.9852**	**0.9852**	**0.9852**	**0.9852**
	100	0.8779	**0.8777**	**0.8777**	**0.8777**	**0.8777**
	1000	0.2202	0.2198	**0.2197**	**0.2197**	**0.2197**
*Bi*_m_ = 10	10	0.9807	**0.9806**	**0.9806**	**0.9806**	**0.9806**
	100	0.8458	0.8455	**0.8454**	**0.8454**	**0.8454**
	1000	0.1480	0.1475	**0.1474**	**0.1473**	**0.1472**

For the substrate concentration in the bulk phase, convergence was achieved, with the results stabilizing to the fourth significant digit when a truncation order of *N* = 30 terms was used. Table 3 presents the results of the convergence analysis for the substrate concentration in the solid phase.

**Table 3:** Convergence Analysis for the Substrate Concentration in the Solid Phase (*C*_S_^*^)

*C*_S_^*^	τ	*N* = 200	*N* = 240	*N* = 280	*N* = 320
*Bi*_m_ = 0.01	10	**0.0009**	**0.0009**	**0.0009**	**0.0009**
	100	**0.0078**	**0.0078**	**0.0078**	**0.0079**
	1000	**0.008**	**0.008**	**0.0081**	**0.0081**
*Bi*_m_ = 0.1	10	**0.1617**	**0.1622**	**0.1625**	**0.1627**
	100	0.1696	**0.17**	**0.1704**	**0.1706**
	1000	**0.0716**	**0.0719**	**0.072**	**0.0721**
*Bi*_m_ = 1	10	**0.7444**	**0.7463**	**0.7477**	**0.7487**
	100	**0.6657**	**0.6675**	**0.6687**	**0.6697**
	1000	**0.1355**	**0.1359**	**0.1361**	**0.1364**
*Bi*_m_ = 10	10	**0.8918**	**0.8942**	**0.8958**	**0.8971**
	100	**0.7736**	**0.7756**	**0.7771**	**0.7781**
	1000	**0.1257**	**0.126**	**0.1263**	**0.1264**

In the solid phase, convergence was achieved with an accuracy of two significant digits, requiring a truncation order of *N* = 240 terms for the substrate. The convergence analysis performed using the MOL indicated that, for the bulk phase, a truncation order of *N* = 30 terms for the substrate was sufficient to ensure numerical stability and accuracy to four significant digits. The tables corresponding to the convergence analyses of the products are provided in Tables S1–S4. Based on these analyses, a truncation order of *N* = 40 terms was adopted for the bulk phase to ensure numerical stability and accuracy to four significant digits. For the solid phase, convergence of the products was achieved with *N* = 340 terms, resulting in an accuracy of two significant digits.

### 3.2 Comparative Analysis of the Adopted Numerical Approaches

The results obtained from the mathematical system defined by eqs 8–20 are compared to evaluate the performance of the different solution approaches in describing the system behavior. The MOL is adopted as the reference solution, providing a basis for assessing the accuracy of the approximate solutions obtained with the classical approximation and CIEA-*H*_1,1_. Figure 5 illustrates the temporal evolution of the volume-averaged dimensionless concentrations of the substrate and products inside the particle for different values of the mass Biot number (*Bi*_m_ = 0.01; 0.1; 1; 10), highlighting the influence of intraparticle diffusion on the reaction performance. It is observed that the different behaviors associated with the *Bi*_m_ values reflect the predominant mass transfer regimes in the heterogeneous system analyzed.

**Figure 5:**
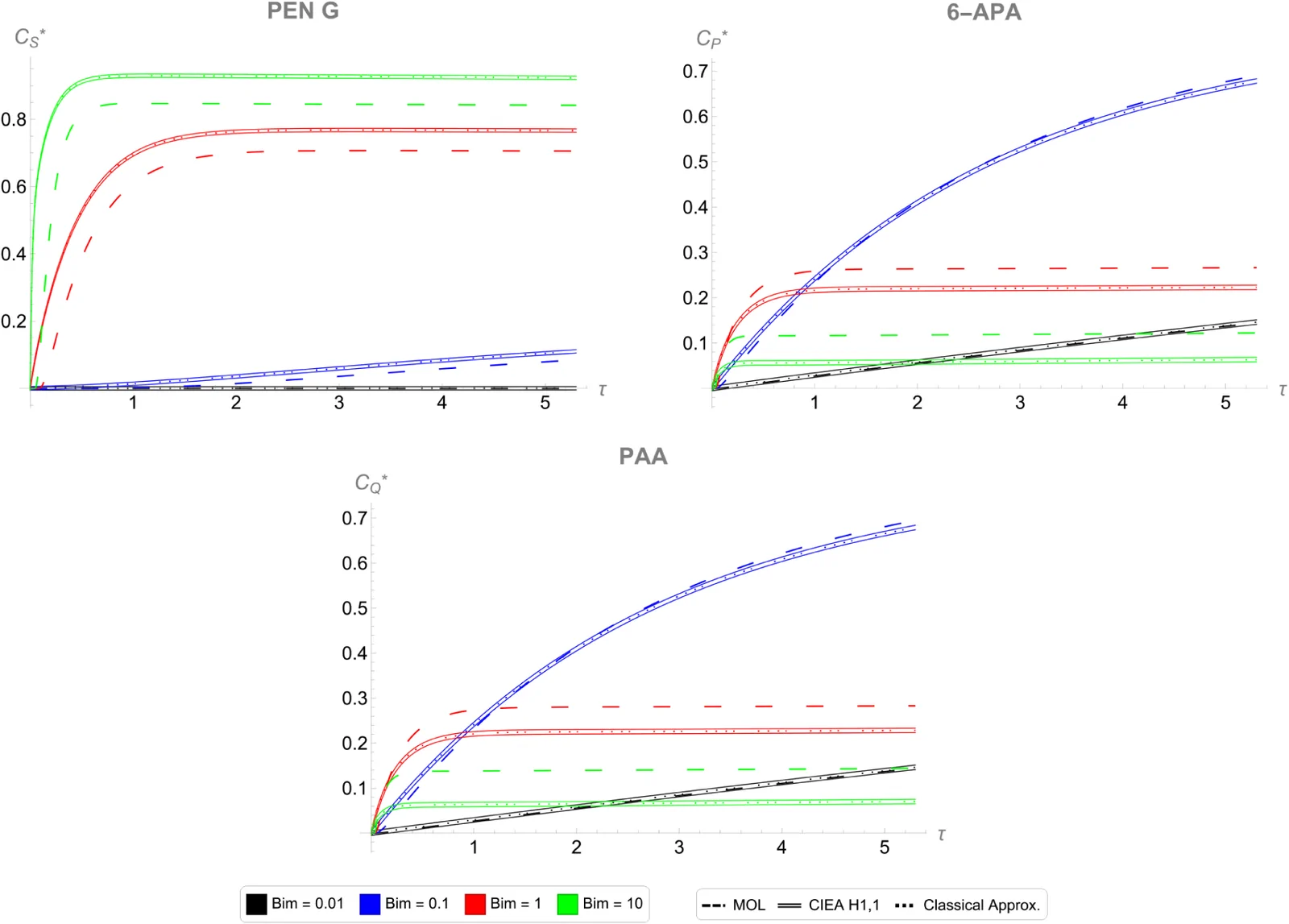
Dimensionless concentration profiles of penicillin G (*C*_S_^*^), 6-APA (*C*_P_^*^), and PAA (*C*_Q_^*^) in the solid phase as a function of dimensionless time (τ) for different *Bi_m_* values, obtained using the classical approximation, the Hermite *H*_1,1_ approximation, and the MOL.

As reported by Al-Muftah and Abu-Reesh,^41^ values of *Bi*_m_ < 0.1 indicate the predominance of external mass transfer resistance, which limits the overall substrate conversion, as also observed in the present results (Figure 5). In contrast, values of *Bi*_m_ > 1 characterize a regime in which internal diffusion resistance becomes dominant, leading to stabilization of the conversion and reduced sensitivity of the system to further variations in *Bi*_m_.

For low *Bi*_m_ values, lower substrate conversion was observed, associated with pronounced concentration gradients inside the immobilized particles, characterizing a system limited by external mass transfer. Conversely, for *Bi*_m_ = 10, faster and more efficient conversion was achieved, indicating that the system operates under an intraparticle diffusion-controlled regime. Thus, analysis of the *Bi*_m_ number provides useful guidance for design decisions, such as adjusting the support porosity, particle geometry, and reactor hydrodynamic conditions, to optimize the efficiency of the enzymatic hydrolysis process.^42^

The solution approach used to solve the mathematical model directly influences the quality of the results, the computational cost, and the ability to adequately represent the physical phenomena involved. The three approaches produced consistent concentration profiles, with close agreement between the classical approximation and CIEA-*H*_1,1_, while small differences were observed relative to the MOL reference solution. The results demonstrate the effectiveness of the approximate solutions in reproducing the behavior predicted by the MOL.

In this context, the CIEA simplifies spatial dependence by applying an averaging procedure along the radial coordinate while preserving the effects of the boundary conditions. This feature makes the method particularly suitable for systems with *Bi*_m_ < 0.1, in which external mass transfer resistance predominates. MOL, in turn, directly solves the system of ordinary differential equations obtained from the spatial discretization of the partial differential equations without resorting to simplifying assumptions regarding the radial profile. In this way, the method preserves the spatial distribution of concentrations and remains valid over the entire range of *Bi*_m_ values, including regimes in which internal mass transfer resistance is significant. Moreover, the MOL allows systematic control of the spatial discretization, enabling mesh refinement to obtain more accurate solutions.

The classical approximation provided a straightforward solution with low computational cost and is applicable in situations in which the radial profile can be represented by simplified functions. The results obtained using this approach exhibited a high level of agreement with the CIEA, particularly for low *Bi*_m_ values, in accordance with the conclusions of Ferreira et al.,^21^ who reported that, in systems with small internal diffusion resistance, averaged solutions can be physically representative. Similar results were reported by Cabral et al.,^23^ who observed compatibility between the classical and *H*_1,1_ approximations in the modeling of microwave-assisted spherical drying.

In this study, substrate conversion increased significantly with the *Bi*_m_ number, reaching maximum values at *Bi*_m_ = 10. This behavior is consistent with the analysis reported by Mapholi et al.,^43^ which indicates that high *Bi*_m_ values are associated with intraparticle diffusion-controlled regimes, in which a detailed spatial representation becomes essential. Under these conditions, the CIEA proves to be an efficient alternative, with the additional advantage of reduced computational cost.

Figure 6 presents a comparison among the three solution methods adopted for the bulk phase. Close agreement is observed between the MOL reference solution and the approximate solutions obtained with the classical approximation and CIEA-*H*_1,1_. For *Bi*_m_ = 0.01, substrate conversion remains limited even at prolonged reaction times, a behavior also reported by Valencia et al.,^44^ who associated this effect with intensified diffusional limitations and local accumulation of inhibitory products. For higher *Bi*_m_ values, particularly *Bi*_m_ = 10, the reduced diffusional resistance allows the substrate to rapidly reach the catalytic sites, resulting in higher conversion rates and increased product formation.

**Figure 6:**
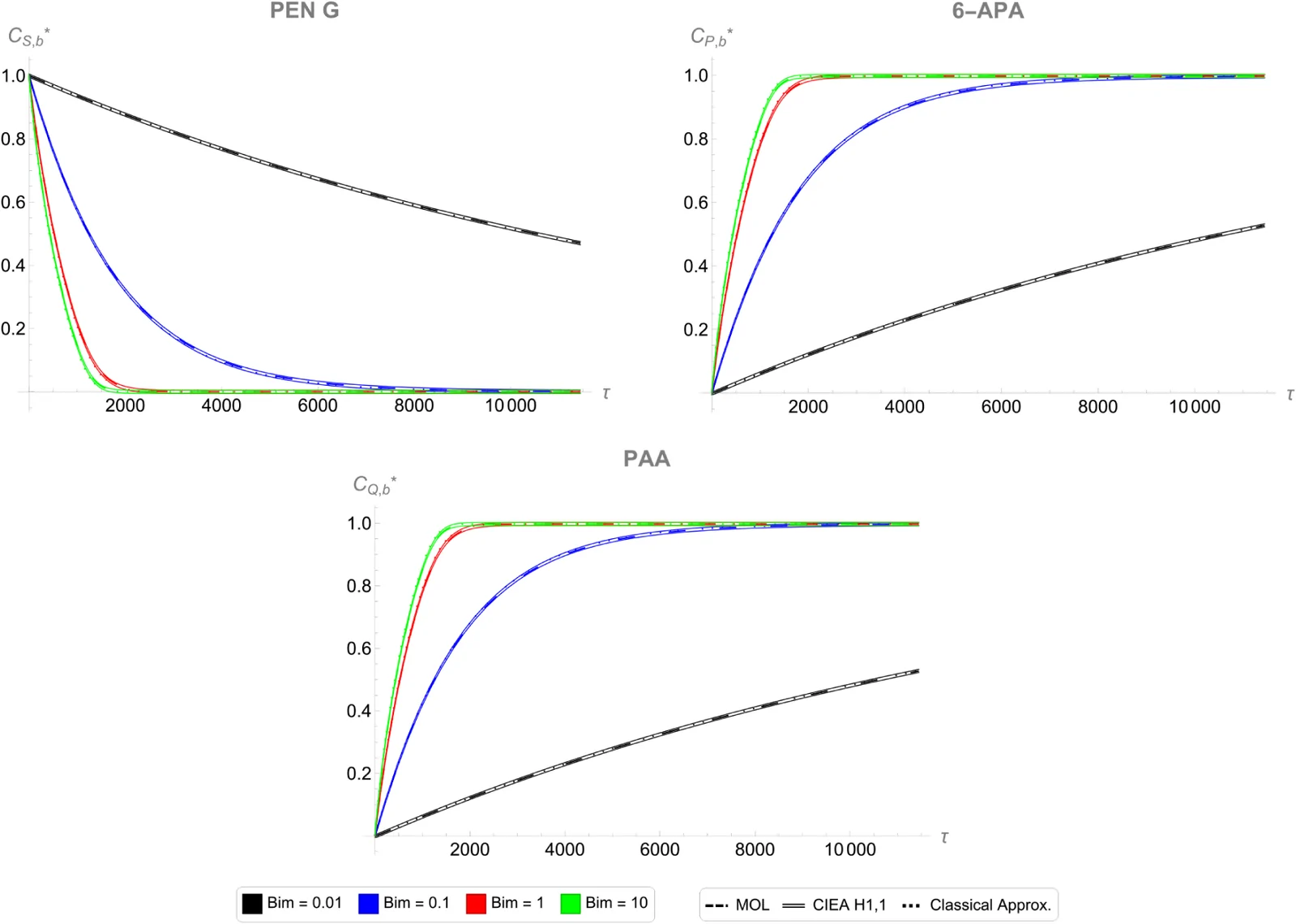
Dimensionless concentration profiles of penicillin (*C*_S,b_^*^), 6-APA (*C*_P,b_^*^), and PAA (*C*_Q,b_^*^) in the bulk phase as a function of dimensionless time (τ) for different mass Biot numbers, obtained using the classical approximation, the Hermite *H*_1,1_ approximation, and the MOL.

The shape of the conversion curves allows identification of the predominant controlling regimes. For *Bi*_m_ = 0.01, an approximately linear profile over time is observed, which is characteristic of systems under strong diffusional limitations. For *Bi*_m_ = 0.1, 1 and 10, the behavior is characterized by a rapid initial increase followed by gradual stabilization, associated with a reduction in convective mass-transfer limitations.^45^

All three solution approaches were effective in solving the proposed problem. The CIEA was advantageous because of its ability to eliminate explicit spatial dependence while preserving the influence of boundary conditions, making it suitable for regimes in which internal diffusion governs the reaction kinetics. The classical approximation also showed good performance even for spherical geometries, as reported by Cabral et al.,^23^ whose results indicated excellent agreement between this methodology and more refined approaches for the temperature field, particularly under low Biot number conditions. The MOL, as discussed by Roknujjaman et al.,^46^ showed high flexibility and accuracy in the numerical solution of partial differential equations by transforming the system into ordinary differential equations that can be solved with high computational efficiency, making it an effective alternative for multidimensional problems with well-defined boundary conditions.^35^

Solving the system using classical approximation and CIEA-*H*_1,1_ involves a significantly lower computational cost than the MOL, which was adopted in this work as the reference solution. In the MOL, the spatial dependence of the PDEs is retained through finite-difference discretization of the radial domain, resulting in a system of ODEs whose dimension increases with the number of grid points. In contrast, the classical approximation and CIEA-*H*_1,1_ provide approximate solutions with a simplified treatment of spatial dependence and, consequently, reduce the number of equations required for the solution, without compromising the accuracy of the results, as demonstrated by their agreement with the solutions obtained using MOL. This reduction in computational cost is particularly relevant for parameter estimation methods that require substantial computational effort, such as the Markov chain Monte Carlo (MCMC) method and the Genetic Algorithm (GA), in which the model must be solved several times.^47–50^ Thus, the use of these reduced and computationally more efficient formulations facilitates the integration of the model with algorithms for estimating kinetic parameters in heterogeneous systems.

## 4 Conclusion

A mathematical model was developed to describe 6-APA production via enzymatic hydrolysis of penicillin G in a heterogeneous system, incorporating the main mass-transfer and reaction mechanisms. The mathematical model was solved using three solution approaches, allowing the resulting solutions to be compared and the agreement among the different approaches in describing the system behavior to be evaluated.

The comparison of the solution approaches showed good agreement between the solutions obtained for both the solid and bulk phases, indicating consistency among the applied approaches. The CIEA exhibited performance comparable to that of classical approximation, while the MOL was adopted as the reference solution and maintained a detailed spatial description of the concentration profiles over the entire range of mass Biot numbers analyzed.

The analysis of the mass Biot number indicated that low values are associated with higher diffusional resistances, whereas high values favor intraparticle mass transfer, leading to higher substrate conversion and product formation. The results provide a solid basis for modeling heterogeneous enzymatic systems and offer useful guidance for the scale-up and optimization of industrial biocatalytic processes.

## Supplementary Material



## Notes

Derivation of the mass balance equations for the bulk and solid phases, eqs S1–S10; the formulation and discretization of the governing equations using the method of lines, including the discretized equations for the products in both phases and their respective initial and boundary conditions, eqs S11–S40; the final equations obtained using the classical and *H*_1,1_ approximations for the products, eqs S41–S90; and the convergence analyses for the bulk and solid phases (Tables S1–S4) (PDF)

## Nomenclature

6-APA: 6-aminopenicillanic acid
7-ADCA: 7-aminodeacetoxycephalosporanic acid
CIEA: coupled integral equations approach
ODEs: ordinary differential equations
PDEs: partial differential equations
MOL: method of lines
MCMC: Markov chain Monte Carlo
GA: genetic algorithm
PAA: phenylacetic acid
PGA: penicillin G acylase

## Symbols

*A*_p_: total particle surface area (m^2^)
*Bi*_m_: mass Biot number
*C*_i_: concentration of species i in the particle
*D*_e_: substrate effective diffusion coefficient (m^2^/s)
*D*_e1_: effective diffusion coefficient of P (m^2^/s)
*D*_e2_: effective diffusion coefficient of Q (m^2^/s)
*D*_i_: effective diffusivity of species i
*D*_r_: reference diffusivity
*C*_P,b_: product P concentration in the bulk phase (mM)
*C*_P_: product P concentration in the particle (mM)
*C*_P,b_^*^: dimensionless concentration of product P in the bulk phase
*C*_P_^*^: dimensionless concentration of product P in the solid phase
*C*_Q,b_: product Q concentration in the bulk phase (mM)
*C*_Q_: product Q concentration in the particle (mM)
*C*_Q,b_^*^: dimensionless concentration of product Q in the bulk phase
*C*_Q_^*^: dimensionless concentration of product Q in the solid phase
*C*_S,b_: substrate concentration in the bulk phase (mM)
*C*_S,b_^0^: initial substrate concentration in the bulk phase (mM)
*C*_S_: substrate concentration in the particle (mM)
*C*_S,b_^*^: dimensionless substrate concentration in the bulk phase
*C*_S_^*^: dimensionless substrate concentration in the solid phase
*C*_E_: enzyme concentration (mM)
*C*_*v*_: Hermite quadrature coefficient associated with the left end point of the interval
*D*_*v*_: Hermite quadrature coefficient associated with the right end point of the interval
*h*_*i*_: interval length between points *x*_*i*–1_ and *x*_*i*_
*I*: average reaction rate
i: chemical species index (S, P, or Q)
*J*: number of spatial discretization subintervals
*k*_1_: rate constant for enzyme–substrate complex formation
*k*_–1_: rate constant for enzyme–substrate complex dissociation
*k*_2_: rate constant for conversion of enzyme–substrate complex into products
*K*: Michaelis–Menten constant for penicillin G (mM)
*K*_1_: inhibition constant for P (mM)
*K*_2_: inhibition constant for Q (mM)
*K*_c_: external mass-transfer coefficient (m/s)
*K*_r_: intrinsic reaction rate constant (1/s)
*K*_S_: substrate inhibition constant (mM)
*R*_i_^*^: volumetric reaction rate for species i (mM/s)
*R*_p_: particle radius (m)
*r*: radial coordinate
*t*: time (s)
*T*_r_: characteristic time (s)
*V*_b_: bulk phase volume (mL)
*V*_tp_: total particle volume (mL)
*v*: derivative order used in the Hermite approximation
*y*_*i*_^(*v*)^: *v*th-order derivative of *y* evaluated at *x*_*i*_

## Greek Symbols

β: derivative order adopted at the right end point in the Hermite approximation
α: dimensionless constant associated with the Michaelis–Menten constant
α_*k*_: derivative order adopted at the left end point in the Hermite approximation
α_S_: dimensionless constant associated with substrate inhibition
α_P_: dimensionless constant associated with inhibition by product P
α_Q_: dimensionless constant associated with inhibition by product Q
γ_S_: dimensionless effective diffusivity of the substrate
γ_P_: dimensionless effective diffusivity of product P
γ_*Q*_: dimensionless effective diffusivity of product Q
η: dimensionless radial coordinate
τ: dimensionless time
φ_part_: particle volume fraction
ψ: Thiele modulus
