# Supplementary material for: Mathematical Modeling of 6‑APA Production by Enzymatic Hydrolysis of Penicillin G in a Batch Reactor
Source: ACS Omega. 2026 Oct 9;11(41):62470–81. doi: 10.1021/acsomega.6c01501 (PMC13689278; doi:10.1021/acsomega.6c01501)
Supplement: Supplementary file 1 [file ao6c01501_si_001.pdf]

Supplementary material for: Mathematical Modeling of 6-APA Production by Enzymatic Hydrolysis of Penicillin G in a Batch Reactor

Letícia Eduarda Alves e Álvares[1], Lucas Figueiredo Formigosa[2], Marcos Vinícius da Silva Paula[3], Mailson Batista de Vilhena[4], Luciana Rocha Barros Gonçalves[5], Emanuel Negrão Macêdo[2], Bruno Marques Viegas[1,2,*]

[1] Graduate Program in Biotechnology, Federal University of Pará, Belém, PA, 66075-110, Brazil.

[2] Graduate Program in Chemical Engineering, Federal University of Pará, Belém, PA, 66075-110, Brazil.

[3] Graduate Program in Materials Science and Engineering, Federal University of Pará, Belém, PA, 66075-110, Brazil.

[4] Department of Production Engineering, State University of Amapá, Macapá, AP, 68900-070, Brazil.

[5] Department of Chemical Engineering, Federal University of Ceará, Fortaleza, CE, 60455-760, Brazil.

E-mail addresses

leaa1702@gmail.com (Letícia Eduarda Alves e Álvares), lucasfigueiredo1227@gmail.com (Lucas Figueiredo Formigosa), mpaula@ufpa.br (Marcos Vinícius da Silva Paula), mailson.vilhena@ueap.edu.br (Mailson Batista de Vilhena), lrg@ufc.br (Luciana Rocha Barros Gonçalves), enegrao@ufpa.br (Emanuel Negrão Macêdo), viegasmbruno@gmail.com (Bruno Marques Viegas).

* Corresponding author.

Mailing address: Graduate Program in Biotechnology, Federal University of Pará, Belém, PA, 66075-110, Brazil.

E-mail address: viegasmbruno@gmail.com (Bruno Marques Viegas).

Mathematical Modeling for the Bulk Phase

Eq. (S1) is a differential equation that accounts for the consumption and formation rates in the system and represents the mass balance in the bulk phase:

$$\frac{dC_i}{dt} = in - out \pm R'' \tag{S1}$$

Eq. (S2) presents the mass balance for the substrate in the bulk phase of the bioreactor, where K_C is the external mass-transfer coefficient.

$$in = K_c \left(C_S\big|_{r=R_p} - C_{S,b} \right) A_p = 0 \tag{S2}$$

Eq. (S3) describes the substrate flux from the bulk phase, accounting for its conversion into products C_P and C_Q, and is given by:

$$out = K_c \left(C_{S,b} - C_S\big|_{r=R_p} \right) A_p \tag{S3}$$

In this model, reactions occur only within the particle; in the bulk phase, the reaction rate is zero. Thus, Eq. (S1) becomes:

$$\frac{dC_{S,b}}{dt} = -out \tag{S4}$$

Rewriting Eq. (S4) and assuming a constant bulk phase volume V_b, Eq. (S5) is derived:

$$\frac{V_b dC_{S,b}}{dt} = -K_c \left(C_{S,b} - C_S\big|_{r=R_p} \right) A_p \tag{S5}$$

where A_p is the total surface area of the particle containing the biocatalyst, as given by Eq. (S6), in which R_P is the particle radius:

$$A_p = \frac{3}{R_p} V_{tp} \tag{S6}$$

In this way, the mass balances for the substrate and products are obtained.

Mathematical Modeling for the Solid Phase

This balance is given by Eq. (S7), representing the concentration changes of the substrate and products.

$$\frac{\partial C_i}{\partial t} = -\nabla \tilde{N}_i \pm R_i" \tag{S7}$$

The flux term is defined by Eq. (S8).

$$\tilde{N}_i = -D_i \nabla C_i + \tilde{u} C_i \tag{S8}$$

Assuming that the convective term is zero and that diffusion occurs only in the radial direction:

$$N_{ir} = -D_i \frac{\partial C_i}{\partial r} \tag{S9}$$

$$\nabla \tilde{N}_i = \frac{1}{r^2}\frac{\partial}{\partial r}\left(r^2 N_{ir}\right) = \frac{1}{r^2}\frac{\partial}{\partial r}\left(r^2 D_i \frac{\partial C_i}{\partial r}\right) \tag{S10}$$

Substituting Eq. (S10) into Eq. (S7) yields the equation describing the concentration changes due to diffusion and reaction.

Method of lines

In this method, the spatial variables are discretized while the temporal variable remains continuous. In Eq. (S11), i represents C_S, C_P and C_Q :

$$\frac{\partial C_i^*}{\partial \tau} = \frac{1}{n^2}\frac{\partial}{\partial n}\left(n^2 \frac{\partial C_i^*}{\partial n}\right) \pm R_i^* \tag{S11}$$

For Eq. (S11), η varies between 0 and 1, with the following initial and boundary conditions:

$$\tau = 0 \rightarrow C_i^* = C_{i,0}^* \tag{S12}$$

$$\eta = 0 \rightarrow \frac{\partial C_i^*}{\partial n} = 0 \tag{S13}$$

$$\eta = 1 \rightarrow -\frac{\partial C_i^*}{\partial n} = Bim_i\left(C_i^* - C_{i,b}^*\right) \tag{S14}$$

Eq. (S15) gives η at a specific point in the spatial domain, allowing precise localization within the computational mesh.

$$\eta_{\text{int}} = \eta_j \tag{S15}$$

Eq. (S16) defines the step size $\Delta\eta$ in the spatial mesh. The step size is inversely proportional to the number of points, so that increasing the number of points decreases the spacing between them.

$$n_j = j\Delta n \tag{S16}$$

Eq. (S17) defines a PDE in which the variation of C_i^* in τ is proportional to the constant R_i^* and factor δ_i. It describes the temporal evolution of C_i^*, incorporating the spatial steps defined in the preceding equations.

$$\frac{\partial C_i^*}{\partial \tau} = \delta_i \pm R_i^* \tag{S17}$$

$$\delta_i = \frac{\gamma_i}{n^2}\frac{\partial}{\partial}\left(n^2 \frac{\partial C_i^*}{\partial n}\right) = \frac{\gamma_i}{n^2}\left\{2n\frac{\partial C_i^*}{\partial n} + n^2 \frac{\partial^2 C_i^*}{\partial n^2}\right\} \tag{S18}$$

$$\delta_i = \frac{2}{n}\frac{\partial C_i^*}{\partial n} + \frac{\partial^2 C_i^*}{\partial n^2} \tag{S19}$$

Discretizing the PDE for $\delta_i = 1 \le j \le n_{j-1}$ at node j of the mesh:

$$\delta_{i,j} = \gamma_i \left\{\frac{2}{j\Delta n}\frac{C_{i,j+1}^* - C_{i,j-1}^*}{2\Delta n} + \frac{C_{i,j+1}^* - 2C_{i,j}^* + C_{i,j-1}^*}{\Delta n^2}\right\} \tag{S20}$$

At node $j = 0$, $\eta = 0$, $\frac{\partial C_i^*}{\partial \eta}$:

$$\left.\frac{\partial C_i^*}{\partial \eta}\right|_{j=0} = \frac{-3C_{i,j}^* + 4C_{i,j+1}^* - 4C_{i,j+2}^*}{2\Delta n} \tag{S21}$$

$$C_{i,j} = \frac{C_{i,j+1}^* - C_{i,j+2}^*}{3} \tag{S22}$$

At node $j = N$, $\eta = 1$, $-\gamma_i \frac{\partial C_i^*}{\partial \eta} = Bim\left(C_i^*\big|_{\eta=1} - C_{i,b}^*\right)$:

$$-\gamma_i \left\{\frac{C_{i,j+2}^* - 4C_{i,j-1}^* + 3C_{i,j}^*}{2\Delta n}\right\} = Bim\left(C_i^*\big|_{\eta=1} - C_{i,b}^*\right) \tag{S23}$$

$$C_{i,j}^* = \frac{Z_i C_i^* + 4C_{i,j-1}^* - C_{i,j-2}^*}{Z_i + 3} \tag{S24}$$

$$Z_i = \frac{2\Delta \eta Bim}{\gamma_i} \tag{S25}$$

For the system, the reaction is expressed as:

$$\tilde{R}_m = 9\psi^2 \frac{C^*_{S,j}}{\left(1+\alpha_S C^*_{S,j} + \alpha\alpha_S (C^*)^2_{S,j} + \alpha_P C^*_{P,j} + \alpha_Q C^*_{Q,j} + \alpha\alpha_Q C^*_{S,j}\theta^*_{Q,j} + \alpha_P\alpha_Q C^*_{P,j} C^*_{Q,j}\right)} \tag{S26}$$

The equations for the substrate and products in the solid phase are given as follows:

$$\frac{dC^*_S}{dt} = \gamma_S\left(\frac{2}{(j-1)\Delta\eta}\frac{C^*_{S,j+1}-C^*_{S,j-1}}{2\Delta\eta} + \frac{1}{\Delta\eta^2}C^*_{S,j+1} - 2C^*_{S,j} + C^*_{S,j-1}\right) - \tilde{R}_m \tag{S27}$$

$$\frac{dC^*_P}{dt} = \gamma_P\left(\frac{2}{(j-1)\Delta\eta}\frac{C^*_{P,j+1}-C^*_{P,j-1}}{2\Delta\eta} + \frac{1}{\Delta\eta^2}C^*_{P,j+1} - 2C^*_{P,j} + C^*_{P,j-1}\right) + \tilde{R}_m \tag{S28}$$

$$\frac{dC^*_Q}{dt} = \gamma_Q\left(\frac{2}{(j-1)\Delta\eta}\frac{C^*_{Q,j+1}-C^*_{Q,j-1}}{2\Delta\eta} + \frac{1}{\Delta\eta^2}C^*_{Q,j+1} - 2C^*_{Q,j} + C^*_{Q,j-1}\right) + \tilde{R}^*_m \tag{S29}$$

The equations for the substrate and products in the bulk phase are given as follows:

$$\frac{dC^*_{P,b}}{dt} = \frac{3Bim\varphi_{part}\left(-C^*_{P,b} + C^*_P\right)}{1-\varphi_{part}} \tag{S30}$$

$$\frac{dC^*_{Q,b}}{dt} = \frac{3Bim\varphi_{part}\left(-C^*_{Q,b} + C^*_Q\right)}{1-\varphi_{part}} \tag{S31}$$

The boundary conditions at $\eta = 0$ are as follows:

$$C^*_{P,0} = \frac{-3C^*_{P,0} + 4C^*_{P,1} - C^*_{P,2}}{2\Delta\eta} = 0 \tag{S32}$$

$$C^*_{Q,0} = \frac{-3C^*_{Q,0} + 4C^*_{Q,1} - C^*_{Q,2}}{2\Delta\eta} = 0 \tag{S33}$$

The boundary condition at $\eta = 1$ are as follows:

$$C^*_{P,Ni} = -\gamma_P \frac{C^*_{P,Ni-2} - 4C^*_{P,Ni-1} + C^*_{P,Ni}}{2\Delta\eta} \tag{S34}$$

$$C^*_{Q,Ni} = -\gamma_Q \frac{C^*_{Ni-2} - 4C^*_{Ni-1} + 4C^*_{Ni}}{2\Delta\eta} \tag{S35}$$

The initial conditions $t = 0$ are as follows:

$$C^*_S = C^*_P = C^*_Q = 0$$
$$C^*_{S,b} = 1; C^*_{P,b} = C^*_{Q,b} = 0 \tag{S36-S40}$$

Classical approach

This approach assumes that radial gradients are small, allowing the surface potential to be approximated by its average values:

$$C_s^* \simeq C_P^* \simeq C_P^*; C_q^* \simeq C_q^* \tag{S41}$$

In addition to this approximation, the term I must be evaluated. To this end, in the integral corresponding to I_i, the local potentials are replaced by their average values. This assumption allows an approximate analytical solution for the term, yielding the equations for the products in the solid phase, given by Eqs. (S42) and (S43).

$$\frac{dC_P^*}{d\tau} = -3Bim\left(C_P^* - C_{P,b}^*\right) - 3I_P \tag{S42}$$

$$\frac{dC_Q^*}{d\tau} = -3Bim\left(C_Q^* - C_{Q,b}^*\right) - 3I_Q \tag{S43}$$

The equations for the products in the bulk phase are given by Eqs. (S44) and (S45).

$$\frac{dC_{P,b}^*}{d\tau} = 3\frac{\varphi_{Part}}{1-\varphi_{Part}} Bim\left(C_{P,b}^* - C_P^*\right)\Big|_{n=1} \tag{S44}$$

$$\frac{d\varphi_{Q_b}}{d\tau} = 3\frac{\varphi_{Part}}{1-\varphi_{Part}} Bim\left(\theta_Q - \varphi_{Q_b}\right)\Big|_{n=1} \tag{S45}$$

Initial conditions:

$$\tau = 0:$$

$$\varphi_{S_b} = \varphi_{S_{b0}};\ \ \varphi_{Q_b} = \varphi_{P_b} = \theta_S = \theta_P = \theta_Q = 0 \tag{S46-S51}$$

Coupled Integral Equation Approach (CIEA)

The first step of this procedure is to determine a solution for the integral, given by:

$$\int_0^1 \frac{\partial\Omega}{\partial\eta} d\eta = \Omega_1 - \Omega_0 = \underbrace{\frac{1}{2}\left[\frac{\partial\Omega}{\partial\eta}\Big|_{\eta=0} + \frac{\partial\Omega}{\partial\eta}\Big|_{\eta=1}\right] + \frac{1}{12}\left[\frac{\partial^2\Omega}{\partial\eta^2}\Big|_{\eta=0} + \frac{\partial^2\Omega}{\partial\eta^2}\Big|_{\eta=1}\right]}_{H_{1,1}\,Approximation} \tag{S52}$$

By substituting the boundary conditions, the following equation is obtained:

$$\Omega_1 - \Omega_0 = \frac{1}{2}\frac{\partial\Omega}{\partial\eta}\Big|_{\eta=1} + \frac{1}{12}\left[\frac{\partial^2\Omega}{\partial\eta^2}\Big|_{\eta=0} + \frac{\partial^2\Omega}{\partial\eta^2}\Big|_{\eta=1}\right] \tag{S53}$$

Applying integration by parts to the integral and using the $H_{1,1}$ approximation, together with the boundary conditions, yields:

$$\int_0^1 \eta^3 \frac{\partial \Omega}{\partial \eta} d\eta = \Omega_1 - \tilde{\Omega} = \underbrace{\frac{1}{2}\left[\left(\eta^3 \frac{\partial \Omega}{\partial \eta}\right)\Bigg|_{\eta=0} + \left(\eta^3 \frac{\partial \Omega}{\partial \eta}\right)\Bigg|_{\eta=1}\right] + \frac{1}{12}\left[\frac{\partial}{\partial \eta}\left(\eta^3 \frac{\partial^2 \Omega}{\partial \eta^2}\right)\Bigg|_{\eta=0} - \frac{\partial}{\partial \eta}\left(\eta^3 \frac{\partial^2 \Omega}{\partial \eta^2}\right)\Bigg|_{\eta=1}\right]}_{H_{1,1}\,Approximation} \tag{S54}$$

Developing mathematical expressions provides an explicit equation for the second derivative, expressed as a function of the average potential, surface area, and flux.

$$\left.\frac{\partial^2 \Omega}{\partial \eta^2}\right|_{\eta=1} = 12\left(\tilde{\Omega} - \Omega_1\right) + 3\left.\frac{\partial \Omega}{\partial \eta}\right|_{\eta=1} \tag{S55}$$

Substituting the corresponding equations gives an expression for the term:

$$\left.\frac{\partial^2 \Omega}{\partial \eta^2}\right|_{\eta=0} = 12\left(\tilde{\Omega} - \Omega_0\right) + 3\left.\frac{\partial \Omega}{\partial \eta}\right|_{\eta=1} \tag{S56}$$

At $\Omega = \theta$, the flux at the boundary is defined by the boundary conditions. The source term $I = 3\int_0^1 \eta^2 \tilde{\mu}(\theta_i(\tau,\eta))d\eta$, can be determined by approximating the local potential with their average values. With this formulation, the system is expressed as follows:

$$\frac{d\tilde{\theta}_P}{d\tau} = -3Bim\left(\theta_P - \varphi_{P_b}\right) - 3I_P \tag{S57}$$

$$\frac{d\tilde{\theta}_Q}{d\tau} = -3Bim\left(\theta_Q - \varphi_{Q_b}\right) - 3I_Q \tag{S58}$$

$$\frac{d\varphi_{P_b}}{d\tau} = 3\frac{\varphi_{Part}}{1-\varphi_{Part}} Bim\left(\theta_P - \varphi_{P_b}\right)\Bigg|_{n=1} \tag{S59}$$

$$\frac{d\varphi_{Q_b}}{d\tau} = 3\frac{\varphi_{Part}}{1-\varphi_{Part}} Bim\left(\theta_Q - \varphi_{Q_b}\right)\Bigg|_{n=1} \tag{S60}$$

$\tau = 0:$

$$\varphi_{S_b} = \varphi_{S_{b0}};\quad \varphi_{Q_b} = 0;\quad \varphi_{P_b} = 0 \tag{S61-S63}$$

$$\theta_S = \theta_P = \theta_Q = 0 \tag{S64-S66}$$

$n = 0:$

$$\frac{d\tilde{\theta}_{S_0}}{dt} = 3\lambda_S \left.\frac{\partial^2 \theta_S}{\partial n^2}\right|_{n=o} - R_i'''\Big|_{n=0} \tag{S67}$$

$$\frac{d\tilde{\theta}_{P_0}}{dt} = 3\gamma_P \frac{\partial^2 \theta_P}{\partial n^2}\bigg|_{n=o} + R_i'''\big|_{n=0} \tag{S68}$$

$$\frac{d\tilde{\theta}_{Q_0}}{dt} = 3\gamma_Q \frac{\partial^2 \theta_Q}{\partial n^2}\bigg|_{n=o} + R_i'''\big|_{n=0} \tag{S69}$$

$$\frac{\partial^2 \theta_S}{\partial \eta^2}\bigg|_{\eta=o} = 3\left(\frac{Bim\left(\varphi_{S_b} - \theta_S\right)\Big|}{\gamma_S} + 4\left(\theta_S - \theta_{S_0}\right)\right) \tag{S70}$$

$$\frac{\partial^2 \theta_P}{\partial \eta^2}\bigg|_{\eta=o} = 3\left(\frac{Bim\left(\varphi_{P_b} - \theta_P\right)\Big|}{\gamma_P} + 4\left(\theta_P - \theta_{P_0}\right)\right) \tag{S71}$$

$$\frac{\partial^2 \theta_Q}{\partial \eta^2}\bigg|_{\eta=o} = 3\left(\frac{Bim\left(\varphi_{Q_b} - \theta_Q\right)\Big|}{\gamma_Q} + 4\left(\theta_Q - \theta_{Q_0}\right)\right) \tag{S72}$$

$n = 1$:

$$\frac{d\tilde{\theta}_{S_1}}{dt} = \left\{2\frac{\partial \theta_S}{\partial n}\bigg|_{n=1} + \frac{\partial^2 \theta_S}{\partial n^2}\bigg|_{n=1}\right\} - R_i'''\big|_{n=1} \tag{S73}$$

$$\frac{d\tilde{\theta}_{P_1}}{dt} = \left\{2\frac{\partial \theta_P}{\partial n}\bigg|_{n=1} + \frac{\partial^2 \theta_P}{\partial n^2}\bigg|_{n=1}\right\} + R_i'''\big|_{n=1} \tag{S74}$$

$$\frac{d\tilde{\theta}_{Q_1}}{dt} = \left\{2\frac{\partial \theta_Q}{\partial n}\bigg|_{n=1} + \frac{\partial^2 \theta_Q}{\partial n^2}\bigg|_{n=1}\right\} + R_i'''\big|_{n=1} \tag{S75}$$

$$\frac{\partial^2 \theta_S}{\partial n^2}\bigg|_{n=1} = -3\left(\frac{Bim\left(\theta_S - \varphi_{S_b}\right)}{\gamma_S} + 4\left[\theta_{S_1} - \theta_S\right]\right) \tag{S76}$$

$$\frac{\partial^2 \theta_P}{\partial n^2}\bigg|_{n=1} = -3\left(\frac{Bim\left(\theta_P - \varphi_{P_b}\right)}{\gamma_P} + 4\left[\theta_{P_1} - \theta_P\right]\right) \tag{S77}$$

$$\frac{\partial^2 \theta_Q}{\partial n^2}\bigg|_{n=1} = -3\left(\frac{Bim\left(\theta_Q - \varphi_{Q_b}\right)}{\gamma_Q} + 4\left[\theta_{Q_1} - \theta_Q\right]\right) \tag{S78}$$

Initial conditions:

$\tau = 0$:

$$C_{S,b}^{*} = C_{S,b}^{*,0};\ C_{Q,b}^{*} = C_{P,b}^{*} = C_{S}^{*} = C_{P}^{*} = C_{Q}^{*} = 0$$
$$C_{S}^{*,0} = C_{P}^{*,0} = C_{Q}^{*,0} = C_{S_1}^{*,0} = C_{P_1}^{*,0} = C_{Q_1}^{*,0} = 0 \quad \text{(S79-S90)}$$

Convergence analysis

Table S1 shows the results of the convergence analysis for product $C_{P,b}^{*}$ in the bulk phase.

Table S1 - Convergence analysis of product $C_{P,b}^{*}$ in the bulk phase.

$C_{P,b}^{*}$	τ	*N=10*	*N=20*	*N=30*	*N=40*	*N=50*
	10	**0.0000**	**0.0000**	**0.0000**	**0.0000**	**0.0000**
Bim = 0.01	100	**0.0044**	**0.0044**	**0.0044**	**0.0044**	**0.0044**
	1000	**0.0615**	**0.0615**	**0.0615**	**0.0615**	**0.0615**
	10	**0.0038**	**0.0038**	**0.0038**	**0.0038**	**0.0038**
Bim= 0.1	100	0.0501	**0.0502**	**0.0502**	**0.0502**	**0.0502**
	1000	0.4222	**0.4223**	**0.4223**	**0.4223**	**0.4223**
	10	**0.0125**	**0.0125**	**0.0125**	**0.0125**	**0.0125**
Bim= 1	100	0.1198	**0.1200**	**0.1200**	**0.1200**	**0.1200**
	1000	0.7775	0.7779	0.7779	**0.7780**	**0.7780**
	10	0.0170	**0.0171**	**0.0171**	**0.0171**	**0.0171**
Bim= 10	100	0.1519	0.1522	**0.1523**	**0.1523**	**0.1523**
	1000	0.8496	0.8502	0.8503	**0.5804**	**0.5804**

Convergence for product $C_{P,b}^{*}$ in the bulk phase was also achieved, albeit more slowly than for the substrate. The solution remained stable up to the fourth significant digit when a truncation order of $N = 40$ terms was used. Table S2 presents the results of the convergence analysis for product $C_{Q,b}^{*}$ in the bulk phase.

Table S2 - Convergence analysis of product $C_{Q,b}^{*}$ in the bulk phase.

$C_{Q,b}^{*}$	τ	*N=10*	*N=20*	*N=30*	*N=40*	*N=50*
	10	**0.0000**	**0.0000**	**0.0000**	**0.0000**	**0.0000**
Bim = 0.01	100	**0.0044**	**0.0044**	**0.0044**	**0.0044**	**0.0044**
	1000	**0.0615**	**0.0615**	**0.0615**	**0.0615**	**0.0615**
	10	**0.0038**	**0.0038**	**0.0038**	**0.0038**	**0.0038**
Bim= 0.1	100	0.0501	**0.0502**	**0.0502**	**0.0502**	**0.0502**
	1000	0.4222	**0.4223**	**0.4223**	**0.4223**	**0.4223**
	10	**0.0125**	**0.0125**	**0.0125**	**0.0125**	**0.0125**
Bim= 1	100	0.1198	**0.1200**	**0.1200**	**0.1200**	**0.1200**
	1000	0.7775	0.7779	0.7779	**0.7780**	**0.7780**
	10	0.0170	**0.0171**	**0.0171**	**0.0171**	**0.0171**
Bim= 10	100	0.1519	0.1522	**0.1523**	**0.1523**	**0.1523**
	1000	0.8496	0.8502	**0.8503**	**0.8503**	**0.8503**

Convergence for product $C_{Q,b}^{*}$ in the bulk phase was also achieved, like product $C_{P,b}^{*}$ with both reaching four significant digits using a truncation order of $N = 40$ terms. Table S3 presents the results of the convergence analysis for product C_{P}^{*} in the solid phase.

Table S3 - Convergence analysis of product C_{P}^{*} in the solid phase.

C_{P}^{*}	τ	*N=200*	*N=240*	*N=280*	*N=320*
	10	**0.2538**	**0.2545**	**0.2549**	**0.2553**
Bim = 0.01	100	0.9258	0.9281	0.9297	**0.931**
	1000	**0.9744**	**0.9768**	**0.9785**	**0.9798**
	10	0.7661	0.768	0.7693	**0.7703**
Bim= 0.1	100	0.9091	**0.8111**	**0.8125**	**0.8136**
	1000	0.9086	**0.9108**	**0.9124**	**0.9136**
	10	**0.2281**	**0.2287**	**0.229**	**0.2293**
Bim= 1	100	**0.308**	**0.3087**	**0.3092**	**0.3096**
	1000	**0.8436**	**0.8457**	**0.8472**	**0.8483**
	10	**0.0775**	**0.0776**	**0.0776**	**0.0777**
Bim= 10	100	**0.1984**	**0.1988**	**0.1991**	**0.1993**
	1000	**0.8536**	**0.8557**	**0.8572**	**0.8584**

Convergence for product C_P^* in the solid phase was also achieved; however, unlike the substrate, a finer mesh was required, as convergence was reached to two significant digits with a truncation order of N = 340 terms. Table S4 presents the results of the convergence analysis for product C_Q^* in the solid phase.

Table S4 - Convergence analysis of product C_Q^* in the solid phase.

C_Q^*	τ	*N=200*	*N=240*	*N=280*	*N=320*
	10	**0.2538**	**0.2544**	**0.2549**	**0.2552**
Bi_m = 0.01	100	0.926	0.9283	**0.93**	**0.9312**
	1000	0.9747	0.9771	0.9788	**0.9801**
	10	0.7683	**0.7702**	**0.7715**	**0.7725**
Bi_m = 0.1	100	**0.8118**	**0.8138**	**0.8152**	**0.8163**
	1000	**0.9102**	**0.9125**	**0.9141**	**0.9153**
	10	**0.2347**	**0.2353**	**0.2356**	**0.2359**
Bi_m = 1	100	**0.3138**	**0.3145**	**0.315**	**0.3154**
	1000	0.846	0.848	0.8496	**0.8507**
	10	**0.0861**	**0.0862**	**0.0863**	**0.0864**
Bi_m = 10	100	**0.2053**	**0.2057**	**0.206**	**0.2063**
	1000	0.8559	0.858	0.8595	**0.8606**

Convergence for product C_Q^* in the solid phase was also achieved, like product C_P^* with both reaching two significant digits using a truncation order of N = 340 terms.
